# Prediction of Atrial Fibrillation Occurrence With Handheld Mobile Electrocardiogram: Deep Learning Model Development Using Real-World Data

**DOI:** 10.2196/87142

**Published:** 2026-08-20

**Authors:** Minje Park, Hyun Jin Ahn, Yeongyeon Na, Sunghoon Joo, Young Ho Lee, Seongwoo Han, Myung Soo Park, Dae Young Cheon, Jeen Hwa Lee, Ki Hong Lee

**Affiliations:** 1 VUNO Inc., Seoul, Republic of Korea; 2 Department of Biomedical Sciences, Chonnam National University Graduate School, Gwangju, Republic of Korea; 3 Department of Cardiovascular Medicine, Chonnam National University Hospital, The Heart Center of Chonnam National University Hospital, 42 Jaebongro, Dong-gu, Gwangju, 61469, Republic of Korea, 82 62-220-6242, 82 62-223-3105; 4 Division of Cardiology, Hallym University Dongtan Sacred Heart Hospital, Hwaseong, Republic of Korea; 5 Department of Internal Medicine, Chonnam National University Medical School, Gwangju, Republic of Korea

**Keywords:** atrial fibrillation, normal sinus rhythm, electrocardiogram, real-world data, mobile health, digital health, AI, deep learning, self-supervised learning, domain adaptation

## Abstract

**Background:**

Atrial fibrillation (AF) is a common arrhythmia associated with an increased risk of stroke and heart failure. To improve prevention, recent studies have used deep learning models to identify at-risk individuals early from normal sinus rhythm (NSR). However, studies using mobile electrocardiogram (mECG) in outpatient, real-world settings remain underexplored.

**Objective:**

The study aimed to develop and evaluate deep learning models using a real-world limb-lead mECG database to predict the short-term occurrence of AF from NSR recordings.

**Methods:**

mECG data were collected from real-world users of commercially available handheld mECG devices capable of capturing 6 limb leads. AF occurrence was defined as an AF event within a predefined time window (7, 14, or 31 d) from the date of the NSR recording. Transformer-based prediction models were developed for limb-lead and lead I input configurations using a multistage training approach with self-supervised pretraining and domain adaptation, drawing on both open, large-scale clinical 12-lead ECG and proprietary real-world mECG databases. The models were evaluated in an internal real-world cohort and explored in an external cohort as a proof of concept via time-to-event analysis.

**Results:**

Between March 2023 and November 2024, 386,519 mECGs were acquired from 8206 users. There were 18,949, 25,206, and 33,524 AF incidences within the 7-, 14-, and 31-day time windows. The models were pretrained with 787,257 12-lead ECGs and 202,689 mECGs, then fine-tuned to predict AF occurrence using 97,447 labeled mECGs. The limb-lead models achieved areas under the receiver operating characteristic curves (AUROCs) of 0.793, 0.785, and 0.787 for 7-, 14-, and 31-day predictions on the internal cohort, respectively, with a user-level AUROC of 0.702 for the 31-day prediction. These models significantly outperformed the lead I models (*P*<.001), supporting the value of multilead configurations. The multistage pretraining was essential, as single-source pretraining yielded lower AUROCs of 0.555 with mECGs only and 0.761 with 12-lead ECGs only for the 31-day prediction. In the subgroup analysis, AUROC values were consistent across age, PR interval, and corrected QT interval, but showed disparities (*P*<.001) by sex (0.713 in females vs 0.794 in males) and by QRS duration (0.583 in ≥120 ms vs 0.796 in <120 ms). In the external cohort (n=144), the 31-day model stratified all 5 new-onset AF events, showing significantly different survival functions between the positively and negatively predicted groups (*P*=.03); Cox proportional hazards regression yielded a hazard ratio of 1.49 (95% CI 1.06‐2.09) per 0.1 increase in model output.

**Conclusions:**

Our findings elucidate the feasibility of deep learning–based AF risk prediction using single-NSR recordings from mobile devices, highlighting the potential for remote AF management in real-world populations. The model output may serve as a risk indicator to support opportunistic AF screening, prompting further clinical evaluation and informing decisions about more intensive monitoring.

## Introduction

Atrial fibrillation (AF) is a common arrhythmia and a major cause of stroke and heart failure. Early identification of individuals at risk is crucial for timely intervention, yet current strategies rely on symptom-driven visits or periodic electrocardiogram (ECG) assessments in clinical settings, which may miss paroxysmal or asymptomatic events.

Mobile electrocardiogram (mECG) devices offer an alternative by enabling user-initiated rhythm monitoring outside clinical environments. Randomized trials have shown that handheld or wearable ECGs improve AF detection compared with standard care [1]. However, most screening methods still depend on directly capturing an AF episode and may miss paroxysmal cases that occur outside the recording window.

Predicting short-term AF risk during normal sinus rhythm (NSR) could enable earlier identification of high-risk individuals. AI algorithms have been increasingly applied to 12-lead and Holter ECGs recorded during NSR, with several studies demonstrating their ability to detect latent signatures indicative of underlying or impending AF [2-9]. More recently, AF prediction has been extended to mECG data collected via smartphone-connected handheld devices and wearable patches [10,11], thereby demonstrating that AI models can estimate near-term AF risk in real-world ambulatory settings. Yet most of these models have been developed and evaluated on single-source datasets, leaving their robustness in independent populations uncertain.

Predicting future AF from NSR recordings is an inherently challenging task, as it requires the model to identify subtle arrhythmogenic signatures hidden within otherwise normal waveforms. This challenge is magnified when using mECGs, which often exhibit greater signal variability and noise artifacts compared with clinical 12-lead ECGs. To overcome these limitations, a robust methodological approach is required. Self-supervised learning (SSL) offers a compelling solution by enabling models to learn powerful, generalizable representations from large pools of unlabeled data [12]. This approach is well-suited for enhancing downstream performance on complex tasks, even with limited annotated samples. Various studies have shown the promise of SSL applications in biomedical time-series tasks, including ECG analysis [13-15]. Furthermore, SSL has been shown to facilitate effective domain adaptation [16,17], allowing models pretrained on one data type (eg, clinical ECGs) to be effectively transferred to another that has different statistical characteristics (eg, mECGs).

In this study, we developed deep learning models that estimate near-term AF risk, potentially useful for aiding clinicians in screening candidates for closer rhythm surveillance, using a real-world mECG database. Specifically, we trained deep learning models to predict AF occurrence within 7, 14, and 31 days following NSR mECGs recorded by a commercially available handheld device. To enhance generalizability, a multistage training pipeline was used as follows: (1) self-supervised pretraining on large-scale clinical ECGs, (2) further pretraining on mECGs for domain adaptation, and (3) supervised fine-tuning for AF prediction. The models were evaluated internally, including subgroup analyses on demographic and ECG-specific variables, and were further tested for new-onset AF risk stratification as an additional proof-of-concept assessment in a clinically distinct external cohort.

## Methods

### Study Design

**Figure 1.**
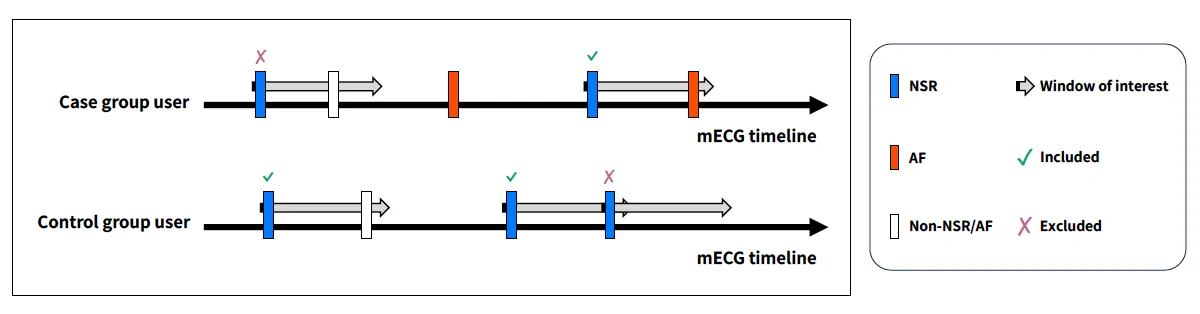
Atrial fibrillation (AF) occurrence labeling and exclusion scheme. Users were classified into a case group or a control group based on the presence of at least one AF recording in the mobile electrocardiogram (mECG) database. For the case group, normal sinus rhythm (NSR) recordings with a subsequent AF recording within a specific time window of interest (eg, 7-d, 14-d, or 31-d) were labeled as AF occurrence positive. For the control group, NSR recordings with a subsequent non-AF recording were labeled as AF occurrence negative. Recordings that did not meet these criteria (eg, NSR without any follow-up recording, NSR followed by non-AF recordings in the case group, or non-NSR ECGs) were excluded from the labeled sets and retained as part of the unlabeled set used for self-supervised pretraining.

We designed a retrospective study to develop and validate deep learning models that predict AF occurrence within short-term windows from a single mECG recorded during NSR. The AF occurrence labeling and exclusion scheme is detailed in Figure 1. Each NSR mECG was considered a potential model input and retrospectively labeled based on the rhythm observed in subsequent recordings within predefined follow-up windows of interest. Labeling was conducted separately for 3 distinct windows: 7, 14, and 31 days. Specifically, an mECG was labeled as positive if AF was detected in any follow-up recordings within the respective window, and negative otherwise. Rhythm classification (eg, NSR or AF) was determined by the device-generated diagnostic output at the time of user-initiated recordings. To be eligible for labeling, an mECG had to be classified as NSR and have at least one follow-up mECG within the corresponding prediction window; all ineligible recordings were excluded from the labeled dataset. Due to this requirement, the labeled sets for 7- and 14-day windows are subsets of the 31-day set, as they exclude recordings that cannot be labeled within the shorter time windows.

### Data Collection

This study used 3 ECG datasets with distinct roles in model development and evaluation: (1) real-world mECGs collected from the general population, (2) a large-scale public 12-lead ECG dataset, and (3) mECGs from individuals without prior AF history but at elevated clinical risk of AF. Each dataset was preprocessed and used according to the specific objectives of model pretraining, training, or testing. No formal a priori sample size calculation was performed; the study used all available recordings.

**Figure 2.**
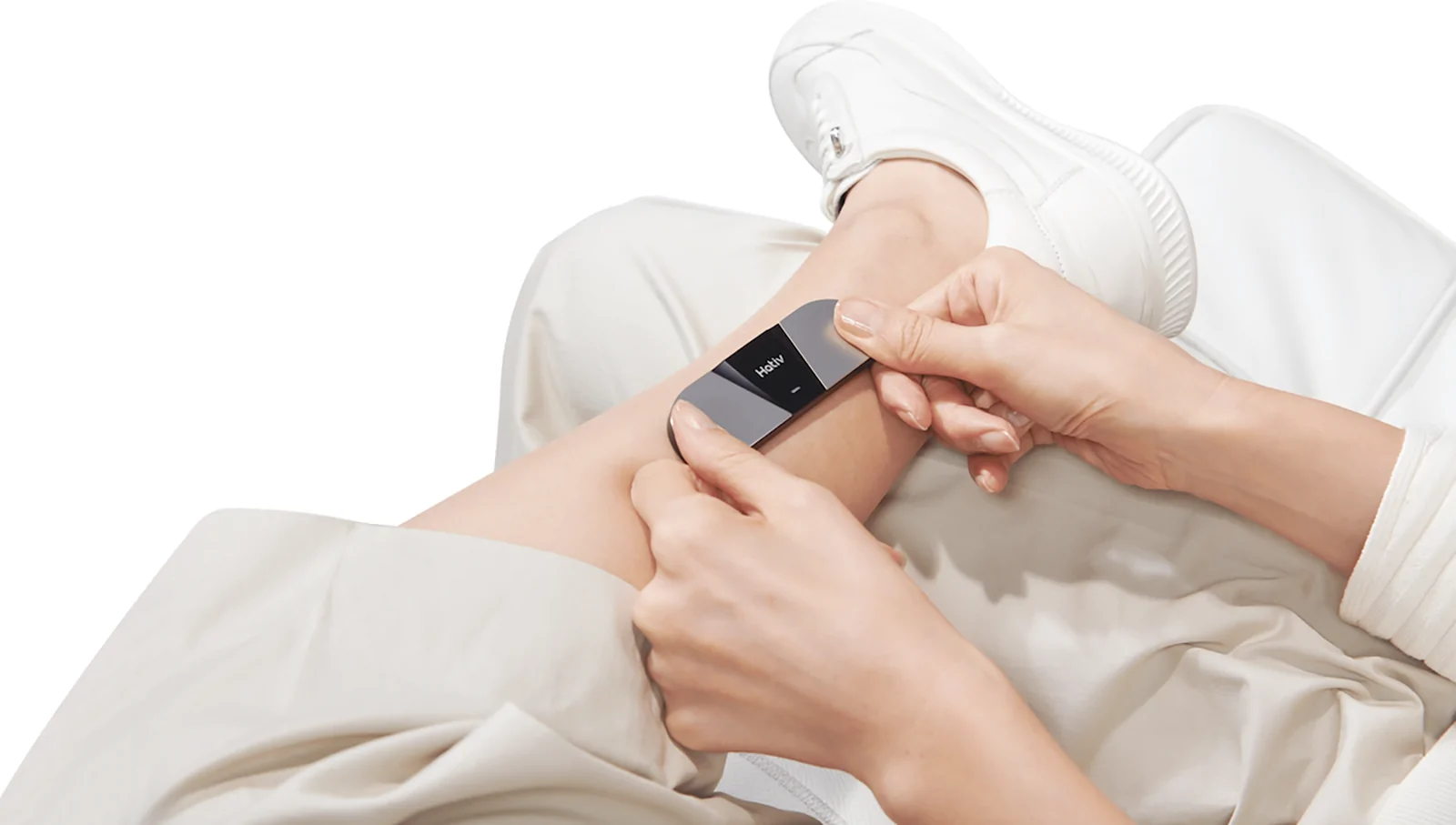
HATIV P30 handheld mobile electrocardiogram device used for data acquisition. The device captures leads I and II directly through 2 electrodes on its top surface, which are held in the user’s hands, and a third electrode on its bottom surface, which is placed on the left leg. The remaining limb leads (III, aVR, aVL, and aVF) are then computationally derived from the measured lead I and II signals using standard transformations. The device performs automatic signal-quality checks during acquisition and prompts the user to rerecord when input quality is inadequate. Validated recordings are then processed by the device’s on-board algorithm for arrhythmia detection, including atrial fibrillation identification. The image is provided courtesy of VUNO Inc.

The first dataset consisted of mECGs recorded from real-world users of HATIV P30 (VUNO Inc; Figure 2), a commercially available handheld mobile device capable of capturing 6 limb leads (I, II, III, aVR, aVL, and aVF). Recordings were collected between March 2023 and November 2024 from real-world device users in South Korea. Only leads I and II were directly measured; the remaining 4 leads were derived using standard transformations. Each mECG was recorded over a 30-second duration, and a clean segment of 15 to 30 seconds was extracted using the signal filtering rule of the device. We excluded mECGs with invalid or missing values to ensure reliable analysis, those classified as noise by the device’s algorithm to maintain adequate signal quality, or recordings with only a single lead to preserve consistent multilead input across the dataset. mECGs were labeled and split at the user level into training, validation, and test sets to prevent user overlap. The training and validation sets were used for supervised learning and model selection, while the test set served for internal performance evaluation. To prevent label noise, mECGs were excluded from the AF labeling process if they were not measured by the device owner (ie, third-party recordings) or were recorded by excessive users. Excessive users were defined as those with an average of 5 or more daily measurements, presumed to be institutional accounts. These excluded recordings were then used only for self-supervised pretraining as part of the unlabeled set. All AF mECGs in the test set were additionally reviewed by a board-certified electrophysiologist with over 10 years of post-board electrophysiology experience at Chonnam National University Hospital, to confirm label quality.

The second dataset was MIMIC-IV-ECG [18], a large-scale public 12-lead ECG database. It consisted of ECGs collected in a clinical setting at Beth Israel Deaconess Medical Center (Boston, United States) from 2008 to 2019, representing a broad spectrum of patients with diverse demographics and cardiac conditions. This dataset was used for large-scale self-supervised pretraining of the model before domain-specific fine-tuning, using only the 6 limb leads or lead I for compatibility with the mECG data.

The third dataset consisted of HATIV P30 mECGs acquired for an ongoing prospective study from patients at Hallym University Dongtan Sacred Heart Hospital who had no prior diagnosis of AF but were stratified as high risk, with a C₂HEST (coronary artery disease or chronic obstructive pulmonary disease, hypertension, elderly, systolic heart failure, and thyroid disease) [19] or HATCH (hypertension, age, transient ischemic attack or stroke, chronic obstructive pulmonary disease, and heart failure) [20] score of ≥4. We used the recordings collected between May 2024 and February 2025. Only the first NSR mECG per patient was available and paired with clinician-reviewed AF incidence, solely to evaluate the model’s risk stratification performance. Enrolled patients were instructed to record mECGs at least weekly and whenever they experienced symptoms, with AF events determined from these recordings. Each AF event in the external cohort, based on review of follow-up mECG waveforms, was confirmed by 1 of 4 board-certified cardiologists at Hallym University Dongtan Sacred Heart Hospital, with a median of 16 (range 10‐33) years of clinical experience.

### Data Preprocessing

All signals underwent a standardized preprocessing pipeline prior to model input. Recordings were resampled to 250 Hz and filtered using a fifth-order Butterworth bandpass filter (0.67‐40 Hz). Distinct segment extraction methods were then applied for training and evaluation. During training, a single, random 9-second segment was cropped from each recording for data augmentation to improve generalizability [21]. This length was selected to approximate the standard 10-second clinical ECG strip while capturing multiple cardiac cycles (≈9‐12 beats at typical heart rates). Each extracted segment was then standardized to have a zero mean and unit variance. During evaluation, each recording was divided into overlapping 9-second segments with a stride of 0.5 seconds. This stride was chosen to provide dense temporal coverage while maintaining computational efficiency during inference. Each segment was individually standardized, and the model’s final prediction was obtained by averaging the outputs across all segments.

### Deep Learning Model Development

The entire deep learning framework was implemented using the *PyTorch* library (PyTorch Foundation). We adopted a multistage training pipeline for developing deep learning models capable of predicting future AF occurrences from NSR. Masked autoencoder (MAE) [13] was used for SSL with a vision transformer [22] as the backbone, motivated by the strong arrhythmia classification performance demonstrated in previous studies [14,15]. The vision transformer takes an ECG signal as input, divides it into sequential segments, and analyzes them through multiple layers using an attention mechanism that weighs how different segments relate to one another, capturing both local waveform features and broader patterns to produce its output. During MAE pretraining, portions of the input ECG signal were randomly masked, and the model learned to reconstruct these hidden portions from the visible remainder, thereby acquiring meaningful representations of cardiac electrical patterns from unlabeled data without requiring outcome labels. An overview of the pretraining framework is illustrated in Figure S1 of Multimedia Appendix 1.

Initial self-supervised pretraining was performed on the MIMIC-IV-ECG limb-lead or single-lead (lead I) signals, enabling the model to learn generalized spatiotemporal ECG representations across a wide range of clinical contexts [14]. To adapt the pretrained model to the mECG domain, we conducted further MAE-based pretraining [16,17] on the mECG pretraining set. This stage aimed to bridge the distributional gap between clinical 12-lead ECGs and recordings from real-world mobile device users.

The pretrained encoder was then fine-tuned using the labeled training set for the supervised AF occurrence prediction task. A balanced focal loss [23] was used to handle class imbalance. We used a strong data augmentation policy using RandAugment [24], which involved a set of transformations inspired by prior ECG augmentation research [25], to improve model generalization and robustness. This was motivated by the inherent difficulty of the task (ie, detecting latent signatures of future AF within NSR recordings) and the noisy nature of recordings collected in real-world settings. The augmentation set used in this study is listed in Table S1 of Multimedia Appendix 1.

AdamW optimizer [26] and a cosine annealing learning rate schedule [27] were used for both the pretraining and fine-tuning stages, and layer-wise learning rate decay [28] was used for fine-tuning. Model selection was performed using the validation set, and the best-performing checkpoint was evaluated on the test set. Hyperparameters were tuned for the 31-day prediction setting; the configurations used in this study are listed in Table S2 of Multimedia Appendix 1.

### Model Evaluation

Model performance was evaluated across 6 separately trained models, defined by combinations of input lead type (limb leads vs lead I only) and prediction window (7, 14, and 31 d). The area under the receiver operating characteristic curve (AUROC) was used as the primary metric. We also reported sensitivity, specificity, positive predictive value (PPV), and negative predictive value (NPV) for greater clinical interpretability. These metrics were calculated using a decision threshold chosen to equalize sensitivity and specificity on the validation set. This balanced threshold reflects equal clinical priorities in the mobile device user setting: early AF detection to enable timely follow-up (sensitivity) and avoidance of false alarms that could cause unnecessary user anxiety in self-monitoring (specificity). We additionally evaluated classification performance at a high-sensitivity threshold (sensitivity=0.9 on the validation set).

We also conducted subgroup analyses of the 31-day prediction model to identify potential performance disparities. Stratified AUROCs were reported by age (<60 vs ≥60 y), sex (male vs female), and key ECG features, including PR interval (<200 ms vs ≥200 ms), QRS duration (<120 ms vs ≥120 ms), and corrected QT interval (QTc; <460 ms vs ≥460 ms). These numeric features were obtained from the device-generated, automated ECG interpretation and were binarized based on clinically defined cutoffs.

For exploratory external evaluation, we applied the trained 31-day limb-lead model to the external cohort. Each patient was assigned a model output and then stratified into a “positively predicted” group or “negatively predicted” group based on the decision threshold previously established on the validation set (where sensitivity equals specificity). We then performed a 31-day time-to-event analysis to compare the cumulative incidence of new-onset AF between these 2 stratified groups. In addition, we estimated the association between the continuous model output and time to incident AF using Cox proportional hazards regression. The model output was entered as a single predictor and scaled per 0.1-unit increase to facilitate clinical interpretation.

### Additional Analyses

We performed 3 sensitivity analyses. First, to assess the contribution of each pretraining stage, we compared three pretraining configurations: (1) MAE pretraining on MIMIC-IV-ECG only, (2) MAE pretraining on mECGs only, and (3) sequential MAE pretraining using MIMIC-IV-ECG followed by mECGs (the default setting in the main experiments). Second, to assess the relative contribution of physically measured versus computationally derived leads, we compared a model trained using only the physically measured leads (leads I and II) against the default configuration using the full set of limb leads (leads I, II, III, aVR, aVL, and aVF). Third, to evaluate model performance independent of within-user contributions, we also performed a user-level analysis, deriving a single per-user prediction by randomly selecting a recording and aggregating outputs. Additionally, we visualized the Transformer model’s attention weights for representative test examples to qualitatively assess model interpretability.

### Statistical Analyses

Statistical analyses were conducted to compare baseline characteristics and evaluate model performance. Baseline demographic and clinical features were compared across the mECG training, validation, and test sets using a one-way ANOVA for continuous variables and the chi-square test for categorical variables. To compare model performance, we used the DeLong method [29] to test for significant differences between AUROCs and to calculate CIs. For sensitivity, specificity, PPV, and NPV, CIs were estimated using the Clopper-Pearson exact method [30]. For the time-to-event analysis in the external cohort, Kaplan-Meier survival curves were generated to estimate the cumulative incidence of new-onset AF in the model-stratified groups, and the difference was tested for significance using the log-rank test. Hazard ratios were reported with 95% Wald CIs. Calibration was assessed using the observed-to-expected (O/E) ratio. A significance level of .05 was used for all analyses. We used Python with open-source libraries, including *SciPy*, *scikit-learn*, *scikit-survival*, and *lifelines*.

### Ethical Considerations

This study was conducted in accordance with ethical standards and was approved by the Institutional Review Board (IRB) of Chonnam National University Hospital (IRB: CNUH-2025‐127). Users provided informed consent for the use of their deidentified data (eg, ECGs, age, and sex) for research purposes during registration on the mobile application platform. The external cohort was collected as part of a prospective observational study registered with the Clinical Research Information Service, Korea (registration: KCT0009304, date of registration: April 5, 2024, IRB: HDT 2023-12-003). In addition, the study used the publicly available MIMIC-IV-ECG v1.0 dataset, which is deidentified and openly accessible via PhysioNet [18]. The MIMIC-IV-ECG project was approved by the IRBs of Beth Israel Deaconess Medical Center and the Massachusetts Institute of Technology, with a waiver of individual patient consent. Formal patient and public involvement was not incorporated, given the retrospective nature of the analysis and the use of pre-existing deidentified data.

## Results

### Study Population and Dataset Split

**Figure 3.**
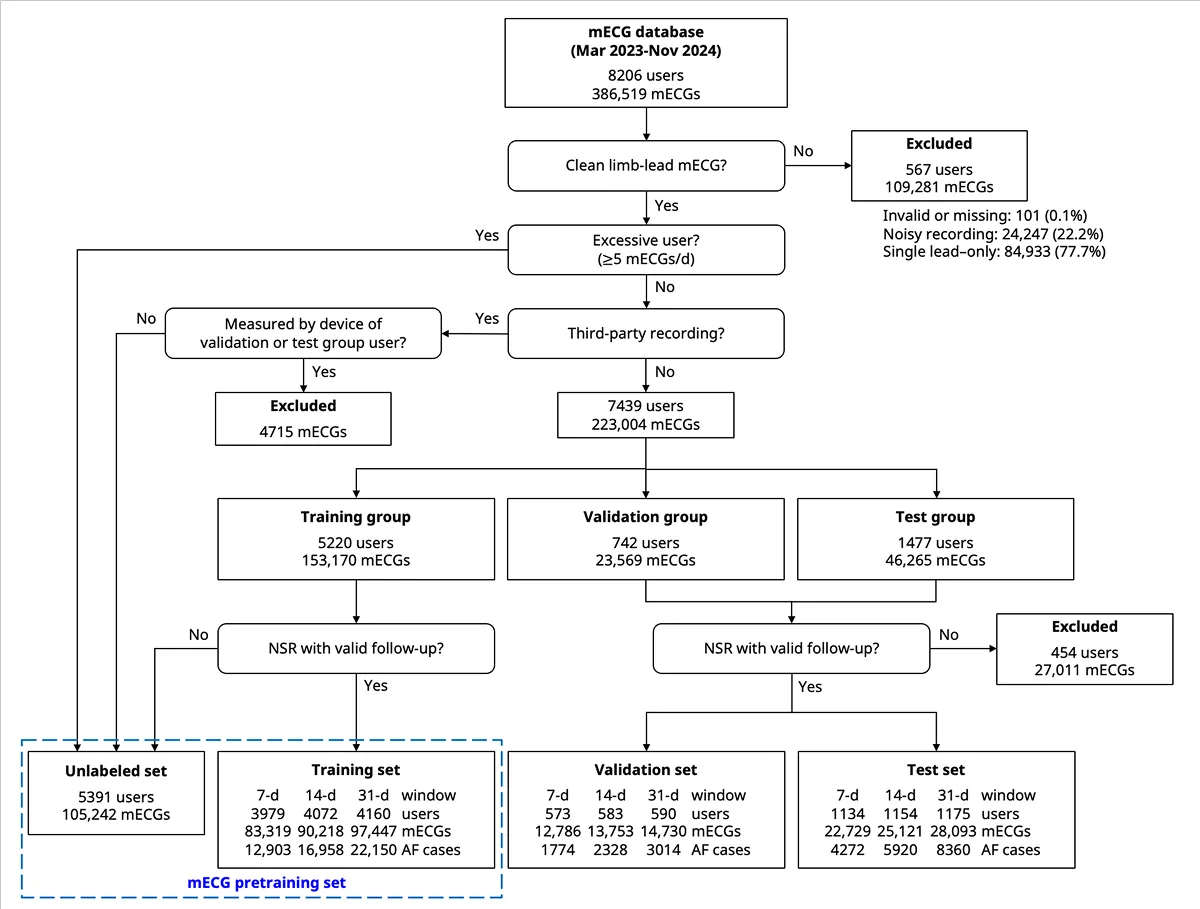
Flow diagram of the mobile electrocardiogram (mECG) dataset construction. Initial data were curated by excluding invalid, low-quality, and single lead–only signals. To prevent data leakage, users were partitioned into training, validation, and test groups. Excessive users (≥5 mECGs/d) and third-party recordings not associated with the validation or test groups were reserved as unlabeled data. Users with at least 1 atrial fibrillation (AF) episode were defined as cases; all others were defined as controls. Final labeled datasets included only normal sinus rhythm (NSR) recordings with valid follow-ups within 31 days: an AF episode for cases and any subsequent recording for controls. The mECG pretraining dataset combined all labeled training data and the unlabeled data.

Between March 2023 and November 2024, 386,519 mECGs were acquired from 8206 mobile device users. Figure 3 illustrates the construction of the mECG dataset. Quality-based exclusions removed 28.3% (109,281/386,519) of mECGs, including 101 with invalid or missing signal values, 24,247 with noise, and 84,933 with only a single lead, leaving 277,238 mECGs. After further setting aside 54,234 recordings from excessive users and third-party users, the cleaned database comprised 223,004 mECGs (7439 users). User‐level stratification yielded training, validation, and test groups containing 153,170 mECGs (5220 users), 23,569 mECGs (742 users), and 46,265 mECGs (1477 users), respectively. After the AF occurrence labeling procedure, the training, validation, and test sets for the 31-day prediction model included 97,447 mECGs (4160 users), 14,730 mECGs (590 users), and 28,093 mECGs (1175 users), respectively, with AF occurrences of 22.7% (22,150/97,447), 20.5% (3014/14,730), and 29.8% (8360/28,093); the numbers of mECGs for the 7- and 14-day prediction models decreased according to the shortened prediction time window and are detailed in Figure 3. Upon review by an electrophysiologist, all AF recordings in the test set were confirmed as AF, with no device-clinician discordance. In addition to these labeled sets, 105,242 mECGs from 5391 users were used as an unlabeled set for mECG pretraining, comprising recordings from excessive users, third-party recordings not measured on devices of users in the validation or test groups, and training group recordings without NSR or valid follow-up. Separately, 1.2% (4715/386,519) third-party mECGs recorded on devices of users in the validation or test groups were excluded entirely to prevent cross-cohort data leakage.

The training, validation, and test sets showed comparable baseline demographics, with no significant differences in mean user age (52.2, 52.1, and 51.6 y, respectively; *P*=.41) or in the proportion of male users (2117/4160, 50.9%; 306/590, 51.9%; and 621/1175, 52.9%; *P*=.48) in the 31-day setting, as shown in Table 1. Meanwhile, AF prevalence differed was variable depending on whether the analysis was at the user level (710/4160, 17.1%; 98/590, 16.6%; and 205/1175, 17.4%; *P*=.90) or the sample level (22,150/97,447, 22.7%; 3014/14,730, 20.5%; and 8360/28,093, 29.8%; *P*<.001). All measured ECG parameters (eg, heart rate, PR interval, QRS duration, and QTc) showed statistically significant differences across the sets (*P*<.001). The 7- and 14-day settings showed similar findings (Tables S3 and S4 in Multimedia Appendix 1).

**Table 1.** Characteristics of mobile ECG training, validation, and test sets in the 31-day prediction setting^a^.

Attributes	Training set	Validation set	Test set	*P* value
Users, N	4160	590	1175	—^b^
Recordings, N	97,447	14,730	28,093	—
Prevalence of AF^c^				
AF users, n (%)	710 (17.1)	98 (16.6)	205 (17.4)	.90
AF recordings, n (%)	22,150 (22.7)	3014 (20.5)	8360 (29.8)	<.001
Demographic variables				
Age (y), mean (SD)	52.2 (13.0)	52.1 (12.7)	51.6 (13.5)	.41
Male, n (%)	2117 (50.9)	306 (51.9)	621 (52.9)	.48
ECG^d^ parameters				
Heart rate, bpm^e^, mean (SD)	72.3 (11.4)	71.0 (11.9)	71.8 (11.3)	<.001
PR interval, ms, mean (SD)	150.7 (21.0)	154.5 (22.7)	149.8 (20.8)	<.001
QRS duration, ms, mean (SD)	88.6 (12.1)	89.7 (12.8)	88.4 (11.2)	<.001
QTc^f^, ms, mean (SD)	407.5 (24.4)	408.7 (22.8)	405.4 (23.2)	<.001

a Continuous variables are summarized as means (SDs) and categorical variables as numbers (%). A one-way ANOVA and a chi-square test were performed to compare the continuous variables and categorical variables, respectively. Demographic statistics were computed at the user level, while statistics for ECG parameters were calculated at the sample level.

b Not available.

c AF: atrial fibrillation.

d ECG: electrocardiogram.

e bpm: beats per minute.

f QTc: corrected QT interval.

The 12-lead ECG pretraining set constructed from the MIMIC-IV-ECG database [18] for multistage training included 787,257 ECGs from 160,771 patients after the exclusion of invalid signals. The external set comprised 144 mECGs from 144 individuals. There were 3.5% (5/144) new-onset AF events that occurred within the 31-day follow-up window in the external set (Table S5 in Multimedia Appendix 1).

### Model Performance

Model performance for the 3 (7-, 14-, and 31-d) time windows is shown in Table 2. For models trained on limb-lead input, the AUROCs were consistent across the time windows: 0.793 (0.785‐0.801) at the 7-day window, 0.785 (0.778‐0.792) at the 14-day window, and 0.787 (0.781‐0.793) at the 31-day window. The narrow CIs indicate that the available sample size provided adequate statistical precision. Using thresholds with equal sensitivity and specificity, the NPV remained above 0.842 across all time windows, while the PPV increased with the prediction window length: 0.432 (0.420‐0.444), 0.481 (0.470‐0.492), and 0.548 (0.538‐0.558), respectively. The results with high-sensitivity thresholds are presented in Table S6 of Multimedia Appendix 1.

**Table 2.** Summary of predictive performance for atrial fibrillation occurrence across 7-, 14-, and 31-day settings^a^.

Time window	AUROC^b^ (95% CI)	Sensitivity (95% CI)	Specificity (95% CI)	PPV^c^ (95% CI)	NPV^d^ (95% CI)
Limb-lead model					
7-day	0.793 (0.785‐0.801)	0.651 (0.636‐0.665)	0.802 (0.796‐0.807)	0.432 (0.420‐0.444)	0.908 (0.904‐0.913)
14-day	0.785 (0.778‐0.792)	0.662 (0.650‐0.674)	0.780 (0.774‐0.785)	0.481 (0.470‐0.492)	0.882 (0.877‐0.887)
31-day	0.787 (0.781‐0.793)	0.659 (0.648‐0.669)	0.770 (0.764‐0.776)	0.548 (0.538‐0.558)	0.842 (0.836‐0.847)
Lead I model					
7-day	0.737 (0.728‐0.746)	0.619 (0.604‐0.633)	0.737 (0.731‐0.744)	0.353 (0.342‐0.364)	0.893 (0.888‐0.898)
14-day	0.727 (0.719‐0.735)	0.586 (0.573‐0.598)	0.753 (0.747‐0.759)	0.422 (0.411‐0.433)	0.855 (0.849‐0.860)
31-day	0.678 (0.671‐0.685)	0.547 (0.536‐0.558)	0.722 (0.716‐0.728)	0.455 (0.445‐0.464)	0.790 (0.784‐0.796)

a The decision thresholds were selected such that sensitivity equaled specificity on the validation set. CIs were computed using the DeLong method for the areas under the receiver operating characteristic curves and the Clopper-Pearson exact method for sensitivity, specificity, positive predictive value, and negative predictive value.

b AUROC: area under the receiver operating characteristic curve.

c PPV: positive predictive value.

d NPV: negative predictive value.

The lead I models consistently showed suboptimal performance compared to the limb-lead models. AUROCs were 0.737 (0.728‐0.746), 0.727 (0.719‐0.735), and 0.678 (0.671‐0.685) for the 7-, 14-, and 31-day time windows, respectively, which are significantly below those of their counterparts trained on limb-lead signals (*P*<.001; Table S7 in Multimedia Appendix 1).

### Subgroup Analyses

Subgroup performance for the limb-lead 31-day prediction model is summarized in Table 3. AUROCs were consistent across subgroups defined by age, PR interval, and QTc. Performance varied by sex, with AUROCs of 0.794 (0.788‐0.801) in male participants and 0.713 (0.698‐0.728) in female participants (*P*<.001). In users with a QRS duration ≥120 ms, AUROC decreased to 0.583 (0.489‐0.677).

**Table 3.** Subgroup analysis of the prediction of 31-day AF occurrence using the limb-lead model^a^.

Group	Samples, n	Prevalence of AF^b^, n (%)	AUROC^c^ (95% CI)	*P* value
Total	28,093	8360 (29.8)	0.787 (0.781‐0.793)	
Sex				<.001
Male	19,554	6855 (35.1)	0.794 (0.788‐0.801)	
Female	8539	1505 (17.6)	0.713 (0.698‐0.728)	
Age (y)				.47
≥60	8564	4012 (46.8)	0.768 (0.758‐0.778)	
<60	19,529	4348 (22.3)	0.763 (0.755‐0.771)	
PR interval (ms)				.25
≥200	345	189 (54.8)	0.755 (0.701‐0.809)	
<200	27,748	8171 (29.4)	0.786 (0.780‐0.792)	
QRS duration (ms)				<.001
≥120	552	60 (10.9)	0.583 (0.489‐0.677)	
<120	27,541	8300 (30.1)	0.796 (0.790‐0.802)	
QTc^d^ (ms)				.66
≥460	370	189 (51.1)	0.796 (0.751‐0.842)	
<460	27,723	8171 (29.5)	0.786 (0.780‐0.792)	

a *P* values assess statistical differences in the AUROCs between subgroups within each category (eg, male vs female). The DeLong method was used to compute the 95% CIs for the AUROCs and the corresponding *P* values.

b AF: atrial fibrillation.

c AUROC: area under the receiver operating characteristic curve.

d QTc: corrected QT interval.

### Additional Analysis Results

Table 4 compares the AF prediction performance across the 3 pretraining configurations. The full sequential pretraining pipeline using MIMIC-IV-ECG followed by mECGs consistently yielded significantly higher performance (*P*<.001) compared to the models pretrained only on mECGs or MIMIC-IV-ECG, which showed AUROCs of 0.761 (0.754‐0.767) and 0.555 (0.548‐0.562) in the 31-day prediction setting. Further sensitivity analysis results, including input lead configuration, user-level evaluation, and attention visualizations are presented in Tables S7 and S8 and Figures S2 and S3 in Multimedia Appendix 1.

**Table 4.** Atrial fibrillation predictive performances under different self-supervised pretraining configurations^a^.

Pretraining set	AUROC^b^ (95% CI)		
	7-day window	14-day window	31-day window
mECG^c^	0.687 (0.679‐0.696)	0.643 (0.635‐0.652)	0.555 (0.548‐0.562)
MIMIC-IV-ECG	0.764 (0.756‐0.772)	0.752 (0.745‐0.760)	0.761 (0.754‐0.767)
MIMIC-IV-ECG→mECG	0.793 (0.785‐0.801)	0.785 (0.778‐0.792)	0.787 (0.781‐0.793)

a Three configurations were considered: (1) mECG dataset alone, (2) MIMIC-IV-ECG alone, and (3) a multistage scheme that first pretrains on MIMIC-IV-ECG and then adapts to mECG data (MIMIC-IV-ECG→mECG). CIs were computed using the DeLong method. Models pretrained with the multistage scheme consistently yielded significantly higher performance (*P*<.001; DeLong test) than those pretrained with single-stage schemes.

b AUROC: area under the receiver operating characteristic curve.

c mECG: mobile electrocardiogram.

### New-Onset AF Incidence Analysis

**Figure 4.**
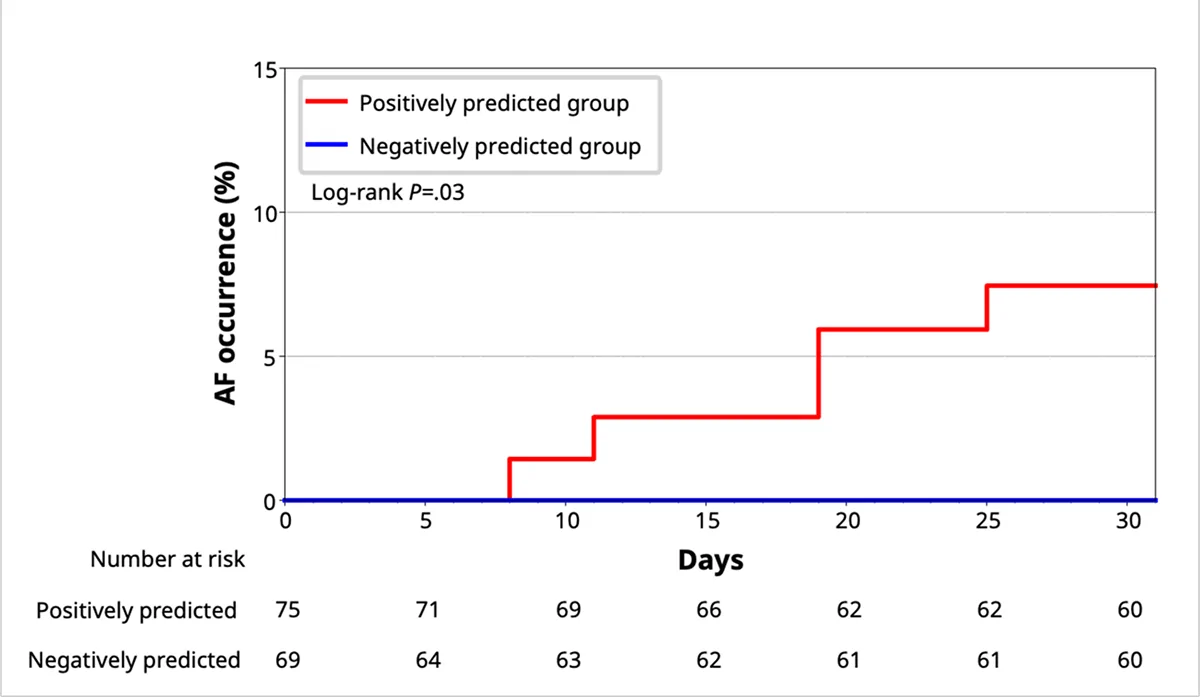
Kaplan-Meier curves for new-onset atrial fibrillation (AF) in the external cohort. The patients were stratified into positively predicted and negatively predicted groups based on the 31-day AF occurrence prediction model applied to each patient’s initial normal sinus rhythm recording, which was collected as part of a prospective study. Survival functions were estimated using the Kaplan-Meier method, based on the time to the first AF occurrence over the 31-day follow-up. The difference in survival distributions was assessed using the log-rank test.

The limb-lead 31-day prediction model was applied to the external NSR cohort with elevated AF-related risk scores. All 5 new-onset AF episodes were in the positively predicted group (5/75, 6.7%); no events were observed in the negatively predicted group (0/69, 0%). Kaplan-Meier survival curves are shown in Figure 4. A significant difference was observed in cumulative AF incidence between the groups (log-rank *P*=.03). Cox proportional hazards regression on the continuous model output (scaled per 0.1-unit increase) yielded a hazard ratio of 1.49 (95% CI 1.06‐2.09; *P*=.02). The O/E ratio for the model outputs was 0.11 (5 observed vs 45.6 expected events), indicating overconfident predictions.

## Discussion

### Model Performance and Comparison With Prior Works

In this study, we developed deep learning models to predict short-term occurrence of AF from mECGs recorded during NSR. Leveraging large-scale, real-world data and a multistage self-supervised pretraining framework, our limb-lead models demonstrated consistent predictive performance across 7-, 14-, and 31-day prediction windows, achieving AUROCs of 0.793, 0.785, and 0.787, respectively. The persistently high performance suggests that our models identify a stable arrhythmogenic substrate rather than transient AF triggers. Notably, these results surpass traditional clinical risk scores, such as the HATCH (AUROC=0.669) and C_2_HEST (AUROC=0.69) [31,32], highlighting the potential of AI-driven mECG analysis to significantly improve AF screening. Furthermore, our results are comparable to a prior study that reported an AUROC of 0.760 for paroxysmal AF prediction from NSR mECG within a 30-day window [11]. The model’s predictions appeared to rely on physiologically meaningful ECG features: visualization of attention weights showed that the model tended to assign the highest weights to P-wave regions, followed by the QRS complex and T-wave regions (Figure S3 in Multimedia Appendix 1), consistent with the P-wave being the ECG signature of atrial electrical activity and the atrial origin of AF.

Because the dataset included multiple mECG recordings per user, we also evaluated the model’s performance at the user level by deriving a single prediction per-user. The user-level AUROC was 0.702 in the 31-day prediction setting, lower than the corresponding recording-level value of 0.787, indicating that recording-level metrics were modestly inflated by within-user contributions. Nevertheless, the user-level performance remained meaningful, supporting the model’s utility for per-user risk stratification. These findings further suggest that aggregating multiple recordings over time may enhance the reliability of individual-level predictions, consistent with the model’s intended use as a serial, opportunistic monitoring tool rather than a single-measurement diagnostic test.

The limb-lead model achieved PPVs of 0.432, 0.481, and 0.548 for the 7-, 14-, and 31-day prediction windows, respectively. While these values could be viewed as indicating that approximately half of positively flagged individuals would not progress to clinically detected AF within the corresponding window, the monotonic increase in PPV with longer prediction horizons is consistent with an early-alarm interpretation: a subset of individuals flagged as positive but not yet meeting the outcome at 7 days may progress to AF over longer intervals. Reported PPVs in AF prediction studies vary widely depending on cohort prevalence, operating point, and prediction window, generally falling in the 0.2 to 0.7 range across studies [2,10,11]. Deployment thresholds can be tuned toward higher PPV when minimizing false positives is prioritized.

While model performance was consistent across age groups (*P*=.47), it differed significantly by sex, with a lower AUROC in the female subgroup (*P*<.001). A similar performance discrepancy between males and females was reported in a previous study of mECG-based AF prediction [11]. This sex-based gap may reflect differences in AF prevalence as well as baseline ECG differences between the sexes. Lower performance was also observed in the wide QRS subgroup with an AUROC of 0.583 (0.489‐0.677), possibly because QRS-widening conditions (eg, bundle branch block, pre-excitation, ventricular pacing) alter the morphology that the model relies on. Model output should be interpreted with caution in these patients, though the small subgroup size (n=552, 60 AF events) and the wide CI limit its reliability; it should be confirmed in larger datasets.

Significant performance differences were observed between the limb-lead and single-lead models (*P*<.001 across all time windows), reaffirming the added value of a multilead configuration even in handheld ECG devices [33]. Sensitivity analysis of input lead combinations in Table S7 of Multimedia Appendix 1 showed that this improvement originated primarily from the inclusion of lead II, reflecting the additional independent information provided by this physically measured channel. The other derived limb leads (III, aVR, aVL, and aVF) contributed only a small additional improvement, likely reflecting their role in supporting model optimization rather than providing additional discriminative information.

When compared to models trained on 12-lead ECGs, our results were relatively modest. For instance, a study by Kim et al [6] achieved AUROCs of 0.812, 0.813, and 0.803 for 7-, 14-, and 28-day AF prediction, respectively, from 12-lead NSR recordings. The performance gaps can be attributed to the richer diagnostic information provided by the precordial leads, which are absent in the mobile setup, as well as the inherent variability and noise present in real-world mobile data compared to the clean, standardized data typically collected in a clinical setting [34,35].

### Effectiveness of Multistage Self-Supervised Pretraining

The effectiveness of our multistage pretraining framework was confirmed through a stepwise analysis. Models trained exclusively on the mECG database had limited predictive ability, achieving an AUROC of 0.555 in the 31-day prediction setting, indicating the difficulty of detecting subtle changes in waveforms related to AF occurrence. Incorporating a large-scale clinical 12-lead dataset (MIMIC-IV-ECG) for initial pretraining led to a substantial performance gain, increasing the AUROC to 0.761. The best performance was achieved with the full sequential approach, pretraining first on MIMIC-IV-ECG and subsequently on the in-domain mECG data, which yielded a final AUROC of 0.787. This stepwise improvement demonstrates the importance of layered representation learning: building foundational knowledge from a large clinical dataset and then refining it through domain adaptation on the mECG data distribution [17].

### Time-to-Event Analysis on an External Cohort

The 31-day prediction model was further evaluated in an external prospective cohort of patients with NSR who had no prior AF, selected on the basis of elevated risk scores (C_2_HEST or HATCH ≥4). The observed stratification of new-onset AF risk suggests that the model retains discrimination in this clinically distinct cohort, even within a population already identified as high-risk by the traditional scores. This highlights the potential of the model for hierarchical application, possibly to identify individuals who require the most intensive surveillance. Given the small number of events observed, however, these findings should be interpreted as preliminary and hypothesis-generating rather than as definitive evidence of external generalizability. An adequately powered evaluation in a larger multicenter cohort is warranted to confirm these initial observations. In addition, the model was developed to optimize discrimination, and its continuous output is most appropriately interpreted as a relative risk score for ranking individuals by AF likelihood. The overconfident predictions in the external cohort (O/E ratio=0.11) are consistent with this discrimination-focused training. Because the external cohort consisted of high-risk patients (C_2_HEST or HATCH≥4) substantially older than the internal cohort (mean 75.1 vs 51.6 y), this overconfidence may have been further amplified. Where absolute event probabilities are required for clinical communication, standard recalibration procedures (eg, Platt scaling, isotonic regression) can be applied as a final step at deployment without retraining the model [36].

### Prevalence of AF in Collected Data

The 17.4% (205/1175) user-level prevalence of AF in the internal cohort is consistent with findings from other large studies of mobile device users and is comparable to the 25.3% (18,661/73,861) prevalence of paroxysmal AF reported by Raghunath et al [11] and the 21.1% (1089/5170) prevalence among patients with a documented history of AF in the work by Kim et al [10]. The high prevalence reflects the user-initiated nature of data collection in real-world settings. Such datasets are often enriched with individuals actively managing a known condition or investigating symptoms, as mobile platforms provide a practical tool for inexpensive, long-term rhythm monitoring necessary for conditions like AF.

The observed 31-day AF incidence of 3.5% (5/144) in the external cohort aligns with previously reported rates in populations with elevated clinical risk. In the REVEAL trial [37], patients with a CHADS_2_ score of ≥3 or ≥2 with additional risk factors had a 6.2% AF incidence within the first 30 days of monitoring using insertable cardiac devices. Similarly, a retrospective study of patients undergoing 30-day ambulatory ECG monitoring reported a 3.4% (78/2326) incidence of newly diagnosed AF among individuals with a comparable risk profile, with a mean (SD) CHA₂DS₂-VASc score of 3.2 (1.8) [38]. Given the nature of our external data, which includes a cohort of individuals with elevated risk scores and follow-up monitoring with self-measured recordings, the observed short-term incidence is clinically plausible. Moreover, the clear separation in AF incidence between positively predicted (5/75, 6.7%) and negatively predicted (0/69, 0%) groups highlights the model’s ability to stratify short-term AF risk even within a population already at elevated risk.

### Clinical Implications

This study marks a conceptual shift from the detection of arrhythmic episodes to the estimation of short-term AF risk using only NSR signals. This approach is particularly valuable because early-stage AF is often paroxysmal and asymptomatic, making it difficult to capture on an ECG even when a patient seeks care for symptoms. Our model addresses this diagnostic challenge by identifying the underlying risk from a readily available NSR recording.

In a real-world clinical pathway, the model’s output could function as a risk score, enabling clinicians to select high-risk individuals for more extensive diagnostic workups, such as long-term continuous monitoring. Given the observed PPV, however, the model’s output should be interpreted as a risk indicator that prompts further evaluation rather than as a standalone AF diagnosis. Interpreting it otherwise could subject patients to unnecessary diagnostic workups, with associated financial costs and anxiety. Because device users access the system for self-monitoring without specialized medical training, they should receive a recommendation to seek follow-up evaluation rather than a definitive diagnostic statement, while clinicians should integrate the model’s output with additional clinical information from the electronic medical record to inform subsequent management decisions [39].

Our study was designed from the outset around an mECG device-user population, and the model’s intended use is correspondingly scoped to this opportunistic monitoring setting rather than general-population screening. While prior studies have focused on identifying silent AF or predicting long-term risk using clinical 12-lead ECGs [2-8], our model leverages mECGs from a commercially available device, supporting the feasibility of short-term, device-based AF risk stratification outside a clinical environment. The time-to-event analysis in the external cohort provides preliminary evidence of the model’s potential as a tool to inform proactive management, although broader generalizability across populations and care settings remains to be established.

### Methodological Considerations

We highlight 3 design choices. First, users with ≥5 mECGs per day were excluded to avoid label noise from potentially shared institutional devices, which could not be distinguished from individual frequent monitoring. However, the excluded subset was small (53 users, 0.7%), making the practical impact minimal, even if some clinically relevant individuals (eg, anxious patients monitoring frequently) were inadvertently removed. Second, third-party recordings retained in the unlabeled pretraining set carry a theoretical risk of overlap with validation or test users, but such overlap is improbable, given the small fraction of these users and the fact that AF labels were not used during pretraining. Third, the labeling strategy depends on AF incidence in future recordings, implying that users with more frequent recordings have a higher likelihood of being categorized as AF-positive. The lower user-level performance (Table S8 in Multimedia Appendix 1) compared with recording-level performance suggests that some surveillance bias may exist. Daily measurement frequency, however, did not differ significantly between AF and non-AF groups, suggesting that frequency-driven bias at the group level may be limited.

### Limitations and Future Work

We acknowledge several limitations in this study. First, we did not have access to detailed clinical information, such as comorbidities relevant to AF development, race, ethnicity, or socioeconomic status, because such sensitive personal information is not captured by the commercial device used for data acquisition. Their absence precluded both adjustment for potential confounding and direct benchmarking against comorbidity-based clinical risk scores. The model’s incremental value over established risk scores, therefore, remains to be determined. Second, outcome labels for the internal cohort relied on device-generated rhythm classifications. Only AF recordings in the test set were reviewed by an electrophysiologist, while the NSR labels and all training and validation set labels were not, resulting in partial verification bias and introducing label noise. Third, in terms of evaluation, the external cohort was insufficient to guarantee adequate statistical power. While the results were significant, they should be interpreted with caution pending confirmation in larger studies. Fourth, the model exhibited sex-based algorithmic bias, with a lower AUROC in the female subgroup, indicating a need for fairness-aware training strategies in future development. Finally, the testing cohorts were limited to users of a commercial mECG device, who likely represent a predominantly Asian population with sufficient purchasing power, potentially introducing selection bias and limiting representation across diverse racial and socioeconomic groups. The exclusion of single lead–only recordings may additionally contribute to this bias if such usage correlates with specific user demographics or behaviors. Furthermore, user-initiated, intermittent recordings may leave asymptomatic AF episodes unrecorded, introducing censoring bias that may inflate apparent specificity and NPV through false-negative labels. Specifically, our labeling convention treated a single non-AF recording within the prediction window as confirmation of AF-free status for the entire window, allowing unrecorded AF episodes to be misclassified as negative.

Future research should address these limitations through several complementary directions. First, integrating model outputs with comorbidity-based clinical risk scores and electronic medical record data may enhance predictive performance and clarify the model’s incremental value over established tools. Second, as the mECG dataset continues to grow, temporal split evaluation using more recently accumulated data will enable the assessment of model robustness against potential temporal drift and evolving user behavior patterns. Third, fairness-aware training strategies, such as sex-balanced sampling or class-weighted loss functions, could help mitigate the observed sex-based performance disparity. Finally, validation in larger and more diverse external cohorts that span racial, ethnic, and socioeconomic backgrounds, ideally with denser monitoring modalities such as continuous patch–based ECG or longer-term Holter recordings, would confirm generalizability, support model fairness across populations, and characterize the impact of user-initiated intermittent sampling and surveillance bias.

### Conclusions

In conclusion, this study successfully developed and validated deep learning models that can predict the short-term occurrence of AF from a single NSR recording obtained with a handheld mECG device. Our models demonstrated robust and consistent predictive performance across 7-, 14-, and 31-day windows and successfully stratified new-onset AF risk in an independent clinical cohort. The effectiveness of a multistage pretraining framework, which leveraged both large-scale clinical and real-world mECG data, was critical to these achievements. These findings highlight the feasibility of using AI-powered mECG analysis for opportunistic, remote screening, shifting the paradigm from arrhythmia detection to proactive risk stratification.

## Supplementary material

10.2196/87142 Multimedia Appendix 1 Supplementary figures and tables supporting the methods and results, including the pretraining framework, model interpretability analyses, experimental settings, cohort characteristics, and additional performance evaluations.

## Abbreviations

AF: atrial fibrillation
AUROC: area under the receiver operating characteristic curve
C₂HEST: coronary artery disease or chronic obstructive pulmonary disease, hypertension, elderly, systolic heart failure, and thyroid disease
ECG: electrocardiogram
HATCH: hypertension, age, transient ischemic attack or stroke, chronic obstructive pulmonary disease, and heart failure
IRB: institutional review board
MAE: masked autoencoder
mECG: mobile electrocardiogram
NPV: negative predictive value
NSR: normal sinus rhythm
O/E: observed-to-expected
PPV: positive predictive value
QTc: corrected QT interval
SSL: self-supervised learning
