# Supplementary material for: Prediction of Atrial Fibrillation Occurrence With Handheld Mobile Electrocardiogram: Deep Learning Model Development Using Real-World Data
Source: JMIR Med Inform. 2026 Aug 20;14:e87142. doi: 10.2196/87142 (PMC13492302; doi:10.2196/87142)
Supplement: Supplementary file 1 [file medinform-v14-e87142-s001.docx]

# Supplemental Materials

## Table of Contents

[Figure S1. Overview of the Self-Supervised Pretraining Framework Based on Masked Autoencoder.	2](#_Toc230483526)

[Figure S2. User-level model output distributions.	3](#_Toc230483527)

[Figure S3. Qualitative Examples for Model Interpretation as an Attention Weight Visualization of the Vision Transformer.	5](#_Toc230483528)

[Table S1. Electrocardiogram Data Augmentations.	6](#_Toc230483529)

[Table S2. Hyperparameter Configurations.	7](#_Toc230483530)

[Table S3. Characteristics of Mobile Electrocardiogram Training, Validation, and Test Sets in 7-Day Prediction Setting.	9](#_Toc230483531)

[Table S4. Characteristics of Mobile Electrocardiogram Training, Validation, and Test Sets in 14-Day Prediction Setting.	10](#_Toc230483532)

[Table S5. Characteristics of External Cohort Set.	11](#_Toc230483533)

[Table S6. Overall Atrial Fibrillation Prediction Performances with High Sensitivity Threshold Selection.	12](#_Toc230483534)

[Table S7. Atrial Fibrillation Prediction Performance: Comparison of Model Variants Across the Prediction Windows.	13](#_Toc230483535)

[Table S8. User-Level 31-day Atrial Fibrillation Prediction Performance of Limb Lead Model	14](#_Toc230483536)

## Figure S1. Overview of the Self-Supervised Pretraining Framework Based on Masked Autoencoder.

The input ECG signal is divided into nonoverlapping patches. A portion of these patches is randomly masked, and only the visible (unmasked) patches are processed by the vision transformer (ViT) encoder to generate latent embeddings. These embeddings are then combined with learnable mask embeddings ([MASK]) at the positions of the original masked patches to form the input for a decoder. The decoder reconstructs the full original signal, and a mean squared error loss is calculated between the reconstructed and original signals, exclusively for the masked patches. The model is optimized to minimize this reconstruction loss.

## Figure S2. User-level model output distributions.

(A) Violin and box plots comparing the user-level mean model outputs between negative (blue) and positive (red) groups in the test set, where Negative denotes users without an AF event during the 31-day follow-up and Positive denotes users with an AF event. Mean, standard deviation (SD), and range of user-level mean model outputs are displayed. Group difference assessed using the Mann–Whitney *U* test. (B) Histogram of per-user standard deviation (SD) of model outputs across multiple recordings, illustrating intra-user variability. The red dashed line indicates the mean intra-user SD (0.086); the green dashed line indicates the inter-user SD (0.174) computed across users for reference. Intra-user variability is approximately half of inter-user variability, indicating relatively consistent model outputs within users.

## Figure S3. Qualitative Examples for Model Interpretation as an Attention Weight Visualization of the Vision Transformer.

Attention weights from the final attention layer of the Vision Transformer were averaged across attention heads, with the CLS token taken as the query. The weights were normalized to the range [0, 1] and overlaid on the corresponding limb lead mECG waveforms as a heatmap, with darker blue shading indicating higher attention weight. The model tended to assign the highest attention weights to P wave regions, followed by QRS complex and T wave regions, consistent with the P wave being the ECG signature of atrial electrical activity and the atrial origin of AF.

## Table S1. Electrocardiogram Data Augmentations.

| **Name** | **Description** |
| --- | --- |
| **Flip** | The signal was randomly flipped horizontally along the time axis. |
| **Drop** | Individual points were randomly selected and set to zero. |
| **Shift** | The entire signal was randomly shifted left or right in time. |
| **Cutout** | A random segment of the signal was masked by setting its value to zero. |
| **Erase** | The entire signal from one randomly selected lead was set to zero. |
| **Sine** | A sine wave was added to the full signal. |
| **PartialSine** | A sine wave was added to a random segment of the signal. |
| **PartialWhiteNoise** | White noise was added to a random segment of the signal. |

## Table S2. Hyperparameter Configurations.

| **Hyperparameter** | **Initial pretraining** | **Subsequent pretraining** | **Supervised fine-tuning** |
| --- | --- | --- | --- |
| **Model configurations** | | | |
| **Encoder backbone** | ViT-Small | ViT-Small | ViT-Small |
| **Patch size** | 25 | 25 | 25 |
| **MLP ratio** | 4 | 4 | 4 |
| **Decoder blocks** | 4 | 4 | N/A |
| **Decoder hidden dimension** | 192 | 192 | N/A |
| **Decoder attention heads** | 6 | 6 | N/A |
| **Masking ratio** | 0.75 | 0.75 | N/A |
| **Optimization configurations** | | | |
| **Optimizer** | AdamW | AdamW | AdamW |
| **Batch size** | 4096 | 4096 | 1024 |
| **Epochs** | 800 | 100 | 100 |
| **LR schedule** | Cosine annealing | Cosine annealing | Cosine annealing |
| **Max LR** | 0.0024 | 0.00015 | 0.004 |
| **Warmup epochs** | 40 | 5 | 5 |
| **Weight decay** | 0.01 | 0.01 | 0.05 |
| **Layer-wise LR decay rate** | 1.0 (no decay) | 1.0 (no decay) | 0.75 |
| **Loss function** | Mean squared error | Mean squared error | Balanced Focal ($\gamma=2, \beta=0.25)$ |
| **Data augmentation** | Random crop | Random crop | Random crop, RandAugment |

MLP, multilayer perceptron; LR, learning rate.

## Table S3. Characteristics of Mobile Electrocardiogram Training, Validation, and Test Sets in 7-Day Prediction Setting.

| **Attributes** | **Training set** | **Validation set** | **Test set** | ***P* value** |
| --- | --- | --- | --- | --- |
| **Users** | 3979 | 573 | 1134 |  |
| **Recordings** | 83,319 | 12,786 | 22,729 |  |
| **AF prevalences** | | | | |
| **AF users** | 623 (15.7%) | 89 (15.5%) | 186 (16.4%) | .82 |
| **AF recordings** | 12,903 (15.5%) | 1774 (13.9%) | 4272 (18.8%) | <.001 |
| **Demographic variables** | | | | |
| **Age, year** | 52.2 ± 13.0 | 52.0 ± 12.8 | 51.5 ± 13.5 | .30 |
| **Male** | 2024 (50.9%) | 296 (51.7%) | 601 (53.0%) | .44 |
| **ECG parameters** | | | | |
| **Heart rate, bpm** | 72.8 ± 11.4 | 71.0 ± 12.1 | 72.2 ± 11.2 | <.001 |
| **PR interval, ms** | 150.3 ± 20.8 | 154.8 ± 22.9 | 149.0 ± 20.3 | <.001 |
| **QRS duration, ms** | 88.1 ± 11.6 | 89.8 ± 13.0 | 88.2 ± 11.4 | <.001 |
| **QTc, ms** | 407.4 ± 24.6 | 409.3 ± 23.1 | 404.9 ± 23.3 | <.001 |

AF, atrial fibrillation; ECG, electrocardiogram; bpm, beats per minutes; ms, milliseconds; QTc, corrected QT interval.

Continuous variables are summarized as mean ± standard deviation and categorical variables as number (%). One-way ANOVA and chi-square test were performed to compare the continuous variables and categorical variables, respectively. Demographic statistics were computed at the user level, while statistics of electrocardiogram parameters were calculated at the sample level.

## Table S4. Characteristics of Mobile Electrocardiogram Training, Validation, and Test Sets in 14-Day Prediction Setting.

| **Attributes** | **Training set** | **Validation set** | **Test set** | ***P* value** |
| --- | --- | --- | --- | --- |
| **Users** | 4072 | 583 | 1154 |  |
| **Recordings** | 90,218 | 13,753 | 25,121 |  |
| **AF prevalences** | | | | |
| **AF users** | 666 (16.4%) | 94 (16.1%) | 195 (16.9%) | .89 |
| **AF recordings** | 16,958 (18.8%) | 2328 (16.9%) | 5920 (23.6%) | <.001 |
| **Demographic variables** | | | | |
| **Age, year** | 52.2 ± 13.1 | 52.0 ± 12.7 | 51.6 ± 13.5 | .41 |
| **Male** | 2076 (51.0%) | 303 (52.0%) | 610 (52.9%) | .51 |
| **ECG parameters** | | | | |
| **Heart rate, bpm** | 72.6 ± 11.4 | 71.0 ± 12.0 | 72.2 ± 11.3 | <.001 |
| **PR interval, ms** | 150.5 ± 20.9 | 154.8 ± 22.8 | 149.3 ± 20.5 | <.001 |
| **QRS duration, ms** | 88.3 ± 11.7 | 89.8 ± 12.9 | 88.3 ± 11.3 | <.001 |
| **QTc, ms** | 407.4 ± 24.5 | 409.1 ± 22.9 | 405.2 ± 23.2 | <.001 |

AF, atrial fibrillation; ECG, electrocardiogram; bpm, beats per minutes; ms, milliseconds; QTc, corrected QT interval.

Continuous variables are summarized as mean ± standard deviation and categorical variables as number (%). One-way ANOVA and chi-square test were performed to compare the continuous variables and categorical variables, respectively. Demographic statistics were computed at the user level, while statistics of electrocardiogram parameters were calculated at the sample level.

## Table S5. Characteristics of External Cohort Set.

| **Attributes** | **External set** |
| --- | --- |
| **Patients** | 144 |
| **AF prevalence** | 5 (3.5%) |
| **Demographic variables** | |
| **Age, year** | 75.1 ± 9.0 |
| **Male** | 84 (58.3%) |
| **ECG parameters** | |
| **Heart rate, bpm** | 72.5 ± 11.7 |
| **PR interval, ms** | 146.4 ± 21.9 |
| **QRS duration, ms** | 88.6 ± 14.9 |
| **QTc, ms** | 409.7 ± 33.6 |

AF, atrial fibrillation; ECG, electrocardiogram; bpm, beats per minutes; ms, milliseconds; QTc, corrected QT interval.

The cohort comprised patients without prior atrial fibrillation (AF) records but with elevated AF risk scores (HATCH or C_2_HEST ≥4). Each patient contributed a single mobile electrocardiogram, the firstly captured normal sinus rhythm recording. AF occurrences were then determined by the 31-day follow-up with the clinician-reviewed diagnosis. Continuous variables are summarized as mean ± standard deviation and categorical variables as number (%).

## Table S6. Overall Atrial Fibrillation Prediction Performances with High Sensitivity Threshold Selection.

| **Time window** | **AUROC (95% CI)** | **Sensitivity (95% CI)** | **Specificity (95% CI)** | **PPV (95% CI)** | **NPV (95% CI)** |
| --- | --- | --- | --- | --- | --- |
| **Limb lead model** | | | | | |
| **7-day** | 0.793 (0.785-0.801) | 0.873 (0.862-0.883) | 0.468 (0.461-0.476) | 0.275 (0.268-0.283) | 0.941 (0.936-0.946) |
| **14-day** | 0.785 (0.778-0.792) | 0.865 (0.856-0.874) | 0.451 (0.444-0.458) | 0.327 (0.320-0.334) | 0.916 (0.910-0.921) |
| **31-day** | 0.787 (0.781-0.793) | 0.892 (0.886-0.899) | 0.418 (0.411-0.425) | 0.394 (0.387-0.401) | 0.902 (0.895-0.908) |
| **Lead I model** | | | | | |
| **7-day** | 0.737 (0.728-0.746) | 0.860 (0.849-0.870) | 0.341 (0.334-0.348) | 0.232 (0.225-0.239) | 0.913 (0.906-0.920) |
| **14-day** | 0.727 (0.719-0.735) | 0.870 (0.861-0.878) | 0.320 (0.313-0.327) | 0.283 (0.276-0.289) | 0.889 (0.881-0.896) |
| **31-day** | 0.678 (0.671-0.685) | 0.858 (0.851-0.866) | 0.277 (0.271-0.284) | 0.335 (0.328-0.341) | 0.822 (0.813-0.831) |

AUROC, area under the receiver operating characteristic curve; CI, confidence interval; PPV, positive predictive value; NPV, negative predictive value.

The decision thresholds were selected such that the sensitivity to be 0.9 with maximizing specificity on the validation set. Confidence intervals were computed using DeLong method for the areas under the receiver operating characteristic curve and the Clopper–Pearson exact method for sensitivity, specificity, positive predictive value, and negative predictive value.

## Table S7. Atrial Fibrillation Prediction Performance: Comparison of Model Variants Across the Prediction Windows.

| **Time window** | **AUROC (95% CI)** |  |  | ***P* value (Full limb leads vs Lead I)** |
| --- | --- | --- | --- | --- |
|  | **Full limb leads (I, II, III, aVR, aVL, aVF)** | **Two leads (I, II)** | **Lead I** |  |
| **7-day** | 0.793 (0.785-0.801) | 0.793 (0.785-0.800) | 0.737 (0.728-0.746) | <.001 |
| **14-day** | 0.785 (0.778-0.792) | 0.773 (0.765-0.780) | 0.727 (0.719-0.735) | <.001 |
| **31-day** | 0.787 (0.781-0.793) | 0.785 (0.779-0.791) | 0.678 (0.671-0.685) | <.001 |

AUROC, area under the receiver operating characteristic curve; CI, confidence interval.

DeLong method was used to compute the confidence intervals of the areas under the receiver operating characteristic curves (AUROCs) and *P* values of AUROC comparisons. Additionally, for the 31-day prediction window, a late-fusion by fitting logistic regression model combining the full limb lead deep learning output with demographic factors (age and sex) yielded an AUROC of 0.780 (95% CI 0.774-0.787), a marginally lower point estimate than the limb lead model (*P*<.001), indicating no improvement in discrimination from adding demographic factors.

## Table S8. User-Level 31-day Atrial Fibrillation Prediction Performance of Limb Lead Model

|  | **AUROC (95% CI)** | **Sensitivity (95% CI)** | **Specificity (95% CI)** | **PPV (95% CI)** | **NPV (95% CI)** |
| --- | --- | --- | --- | --- | --- |
| **All recordings** | 0.787 (0.781-0.793) | 0.659 (0.648-0.669) | 0.770 (0.764-0.776) | 0.548 (0.538-0.558) | 0.842 (0.836-0.847) |
| **One recording per user** | 0.702 (0.662-0.742) | 0.507 (0.437-0.578) | 0.764 (0.736-0.790) | 0.312 (0.263-0.365) | 0.880 (0.856-0.901) |

AUROC, area under the receiver operating characteristic curve; CI, confidence interval; PPV, positive predictive value; NPV, negative predictive value.

For user-level performance evaluation, single recording was randomly sampled for each user. To compute confidence intervals, DeLong method and Clopper–Pearson exact method was used for the areas under the receiver operating characteristic curves (AUROCs) and other metrics (sensitivity, specificity, positive predictive value, negative predictive value), respectively. To assess potential surveillance bias, we additionally compared the average daily mECG measurement frequency between case and control groups. Median (interquartile range, IQR) was 0.48 (0.97) for case group and 0.62 (0.83) for control group, without significant difference (Mann–Whitney *U* test, *P*=.11). However, the lower user-level performance compared with the recording-level performance indicates partial inflation of recording-level metrics by multiple recordings per user, and the possibility of surveillance bias cannot be fully excluded.
